# Predicting Diagnostic Gene Biomarkers Associated With Immune Checkpoints, N6-Methyladenosine, and Ferroptosis in Patients With Acute Myocardial Infarction

**DOI:** 10.3389/fcvm.2022.836067

**Published:** 2022-02-11

**Authors:** Xiao Tong, Xinyi Zhao, Xuan Dang, Yan Kou, Junjie Kou

**Affiliations:** ^1^Department of Cardiology, The Second Affiliated Hospital of Harbin Medical University, Harbin, China; ^2^Key Laboratory of Myocardial Ischemia, Chinese Ministry of Education, Harbin, China

**Keywords:** acute myocardial infarction, differentially expressed genes, ferroptosis, immune checkpoints, m6A, diagnostic gene biomarker

## Abstract

This study aimed to determine early diagnosis genes of acute myocardial infarction (AMI) and then validate their association with ferroptosis, immune checkpoints, and N6-methyladenosine (m6A), which may provide a potential method for the early diagnosis of AMI. Firstly, we downloaded microarray data from NCBI (GSE61144, GSE60993, and GSE42148) and identified differentially expressed genes (DEGs) in samples from healthy subjects and patients with AMI. Also, we performed systematic gene ontology (GO) and Kyoto Encyclopedia of Genes and Genomes (KEGG) analyses and used STRING to predict protein interactions. Moreover, MCC and MCODE algorithms in the cytoHubba plug-in were used to screen nine key genes in the network. We then determined the diagnostic significance of the nine obtained DEGs by plotting receiver operating characteristic curves using a multiscale curvature classification algorithm. Meanwhile, we investigated the relationship between AMI and immune checkpoints, ferroptosis, and m6A. In addition, we further validated the key genes through the GSE66360 dataset and consequently obtained nine specific genes that can be used as early diagnosis biomarkers for AMI. Through screening, we identified 210 DEGs, including 53 downregulated and 157 upregulated genes. According to GO, KEGG, and key gene screening results, *FPR1, CXCR1, ELANE, TLR2, S100A12, TLR4, CXCL8, FPR2* and *CAMP* could be used for early prediction of AMI. Finally, we found that AMI was associated with ferroptosis, immune checkpoints, and m6A and *FPR1, CXCR1, ELANE, TLR2, S100A12, TLR4, CXCL8, FPR2* and *CAMP* are effective markers for the diagnosis of AMI, which can provide new prospects for future studies on the pathogenesis of AMI.

## Introduction

Coronary heart disease affects 17.1 million people worldwide and results in a considerable number of fatalities, making it a global health concern (1). Acute myocardial infarction (AMI) is one of the most serious ischemic heart diseases caused by the rupture of atherosclerotic plaque (2–4). Early and correct diagnosis may be of great benefit in treatment (5–7). Previous studies have identified several risk factors associated with the onset of AMI, including age, gender, hypertension, diabetes, smoking, alcohol consumption, and physical labor (8–11). There is growing evidence that genetic factors contribute to the development of AMI (12). In recent years, various therapeutic targets for AMI have been identified through the study of genetic factors such as mRNA (13–16). Therefore, it is necessary to explore new biomarkers with high sensitivity and specificity for the diagnosis of cardiovascular disease.

In recent years, the development of microarray technology has allowed the identification of biomarkers for diagnosis and prognosis through differentially expressed genes (DEGs) (16, 17). Some ncRNAs can serve as biomarkers for many cardiovascular diseases (18–20), including AMI (mir-1, mir-133, mir-208, and mir-499) (21, 22), acute coronary syndrome (mir-208a, mir-34a, mir-133a, and mir-499) (23), and heart failure (mir-499, mir-133, mir-423-5p, and mir-126) (24–28). Thus, these stable, conserved, and specific RNAs may provide a novel approach to diagnosing cardiovascular diseases.

Herein, we downloaded the microarray data from NCBI, identified DEGs in AMI samples, and compared them with normal controls (29). The identification of DEGs was followed by systematic GO and KEGG analyses (30–33). Protein–protein interactions (PPIs) among the products of DEGs were studied using STRING (34, 35). MCC and MCODE algorithms in cytoHubba plug-in were used to screen 9 key genes in the network. In order to further confirm the stability of these genes, we also confirmed in the GSE66360 dataset, and finally identified 9 key genes. Finally, the genes were identified and examined to determine if these genes with AMI were associated with immune checkpoints, ferroptosis, and N6-methyladenosine (m6A) modification (33, 36, 37). In conclusion, this study provides new insights into the molecular mechanisms responsible for the occurrence of AMI.

## Materials and Methods

### Microarray Data

From the GEO database^1^, we used the MiniML microarray dataset (GSE42148, GSE60993, GSE66360 and GSE61144). The GSE42148 dataset was based on a GPL13607 platform Agilent-028004 SurePrint G3 Human GE 8x60K Microarray (Feature Number Version). GSE60993 dataset was based on Illumina HumanWG-6 V3.0 Expression Beadchip of the GPL6884 platform. GSE61144 dataset was based on Sentrix Human-6 V2 Expression BeadChip of the GLP6106 platform. GSE66360 dataset was based on the GLP 570 [HG-U133_Plus_2] Affymetrix Human Genome U133 Plus 2.0 Array. We included peripheral blood from the patients with ACS who visited the emergency department within 4 h after the onset of chest pain: a set of blood samples of patients with STEMI (*n* = 7) and normal control (*n* = 10) in GSE61144. Enrolled peripheral blood from the patients with ACS who visited the emergency department within 4 hours after the onset of chest pain: ST-elevation myocardial infarction (STEMI, *n* = 7), Non-ST-elevation MI (NSTEMI, *n* = 10) and normal control (*n* = 7) in GSE60993. And we selected the blood samples of myocardial infarction (MI, *n* = 6) patients and healthy control (*n* = 11) in GSE42148. Based on platform annotation information, we converted the probes into gene symbols through the Strawberry Perl language (version 5.32.1.1), and excluded probes containing multiple genes. Furthermore, we removed the batch effect using sva packing in R (38).

### Filtering DEGs

We identified differential expression of RNAs using the “Limma” package in R. There were 30 AMI cases and 28 healthy controls. We then analyzed the adjusted *P-*values to correct the false positive results in the GEO dataset. The adjusted *P-*value <0.05 and | log2 fold-change (FC) | > 1.5 represented the statistical standards for RNA expression screening. We obtained a box graph using the R package GGplot2. The R packages ggord and pheatmap were used to draw the PCA diagram and heatmap, respectively. The above analysis methods were implemented using R Foundation of Statistical Calculation (2020) version 4.0.3 (38, 39).

### Functional Enrichment Analysis

We used GO for functional gene annotation, particularly annotating molecular function (MF), biological pathways (BP), and cellular components (CC). The KEGG enrichment analysis provided a good reference for gene function research and the correlating genomic functional information. To have a better understanding of the effect caused by mRNAs, we applied the ClusterProfiler package (version: 3.18.0) in R to analyze GO functions of potential targets and the KEGG pathway enrichment (40).

### Screening of Candidate Diagnostic Biomarkers

The interactive gene retrieval tool, STRING, is an online biological database that provides gene analysis and builds gene interaction networks at the protein level (41). In this study, we constructed the protein–protein interaction network of DEGs using STRING (Version 11.0) (33, 41). We then visualized the PPI network using Cytoscape version 3.8.2 (34, 36). MCC and MCODE algorithms in cytoHubba plug-in were used to screen key genes in the network.

### Diagnostic Value of Characteristic Biomarkers in AMI

In order to test the predictive value of identified biomarkers, we used the GLM function in R (version 3.6.3) package to build logistics model, and used the GGploT2 package to visualize the results. Receiver operating characteristic (ROC) curves were generated using the mRNA expression data from the GSE42148, GSE60993, and GSE61144 datasets. Data of 30 patients with AMI and 28 patients without AMI was available. The diagnostic values of the identified hub genes were evaluated using the area under the ROC curve (AUC), which was between 0.5 and 1. The closer the AUC is to 1, the better is the diagnostic effect. AUC ranging from 0.5 to 0.7 indicates a low degree of accuracy, while the accuracy of AUC ranging from 0.7 to 0.9 is greater. When the AUC value is >0.9, the accuracy is the highest.

### Effect of the Immune Checkpoint-, m6A-, and Ferroptosis-Related Gene Expression in AMI

Based on the results of previous studies, we identified immune checkpoint-, ferroptosis-, and m6A-related genes. The dataset we downloaded was from the GEO database and the data format was MiniML. We obtained the expression of immune checkpoint-related genes. To derive ferroptosis-related genes, we used the systematic analysis of the aberrances and functional implications of ferroptosis in cancer published by Liu et al. (42). We used the molecular characterization and clinical significance of m6A modulators across 33 cancer types published by Juan Xu to derive the m6A-related genes (43). In addition, we carried out multi-gene Spearman correlation analysis on the immune checkpoint, ferroptosis and m6A methylation to describe the correlation between immune checkpoint, ferroptosis and m6A genes, respectively. And the value of *P* (<0.05) was considered to be statistically significant. Besides, box plots, PCA graphs, two-gene correlation graphs, and multi-gene correlation graphs were obtained by boxplot, R software package ggord, R software package ggstatsplot, and R software package pheatmap, respectively. All of the above analysis methods were implemented using the R foundation for statistical computing (2020) version 4.0.3 (43, 44).

### Validation of Diagnostic Genes

To further validate the genes obtained in our study, we downloaded an additional set of acute myocardial infarction data (GSE number: GSE66360) from the GEO database, including 50 AMI patients and 49 healthy subjects. We performed a log2 transform of the obtained data and subsequently examined the relative gene expression levels of the transiformed data corresponding to the healthy subjects and AMI patients using wilcoxon rank sum test. The values of *P* (<0.05) proved that there were significant differences in gene expression levels between healthy subjects and AMI.

## Results

### Identification of Differentially Expressed Genes

**Figure 1:**
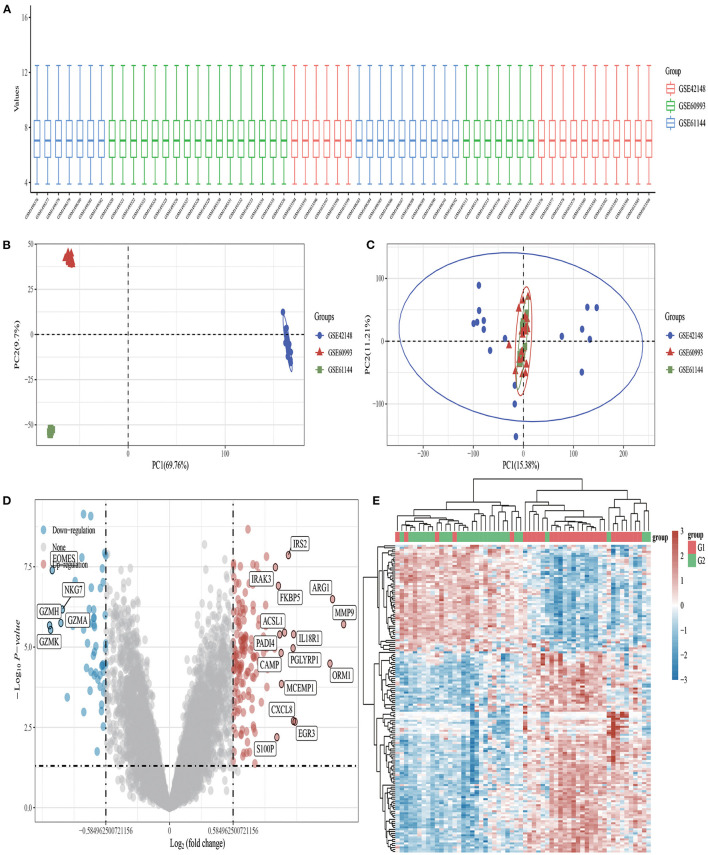
Volcano plots and heat maps of differentially expressed genes in AMI. **(A)** Box plot after data standardization. **(B)** PCA results before batch removal for multiple data sets. **(C)** PCA results after batch removal. **(D)** Volcano plots were constructed using fold-change values and adjusted *P*. The red point in the plot represents the upregulated mRNAs and the blue point indicates the downregulated mRNAs with statistical significance. **(E)** Hierarchical clustering analysis of mRNAs, which were differentially expressed between patients with AMI and healthy controls.

DEGs in GSE42148, GSE60993, and GSE61144 datasets were identified using Limma quartile normalization and background correction methods. Limma screening identified 210 DEGs, including 53 downregulated and 157 up regulated genes (Figure 1).

### Functional Correlation Analysis

**Figure 2:**
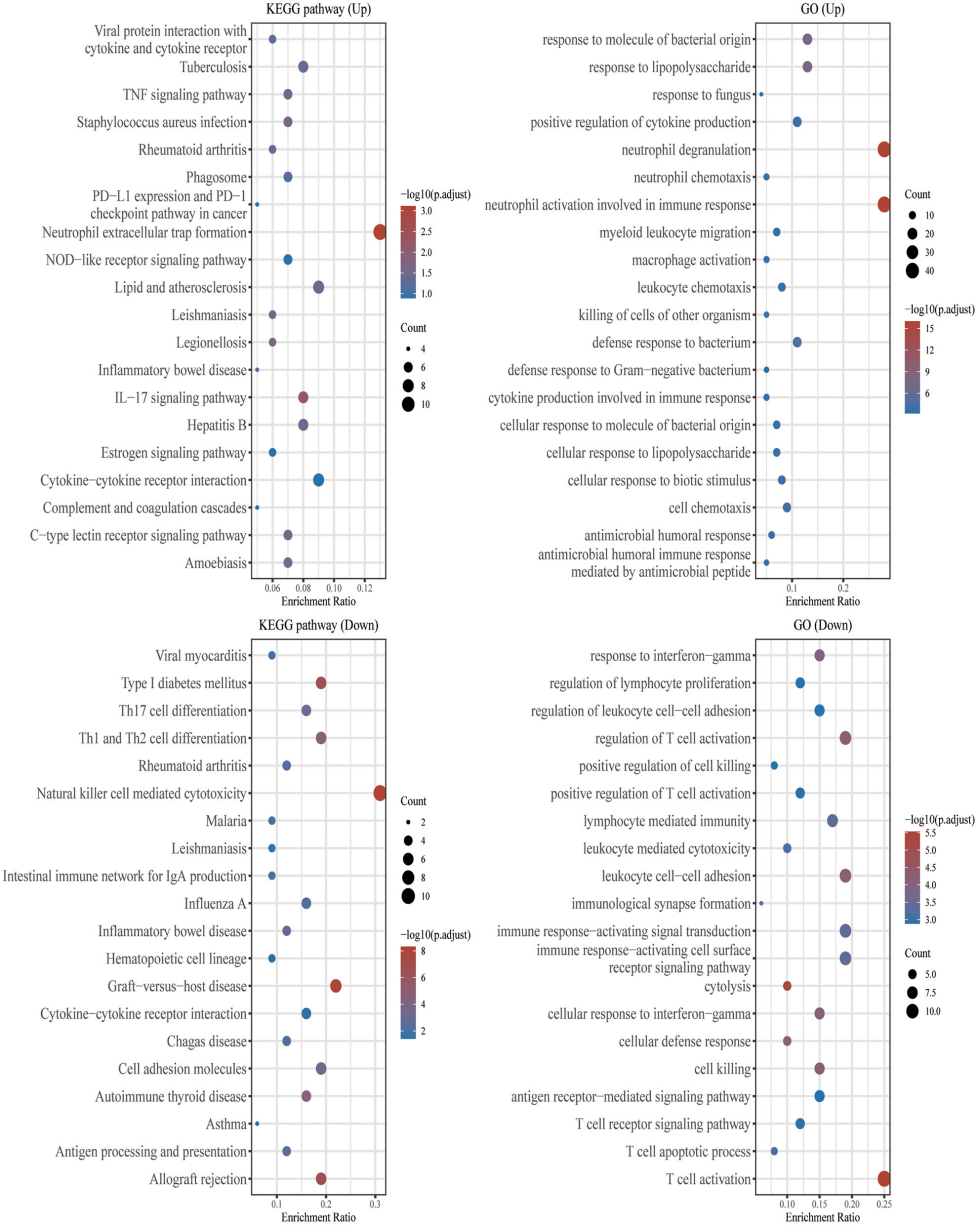
GO and KEGG enrichment analysis. The enriched KEGG signaling pathways were selected to demonstrate the primary biological actions of major potential mRNAs. The abscissa indicates gene ratio and the enriched pathways were presented in the ordinate. Gene ontology (GO) analysis of potential mRNA targets. The biological pathways (BP), cellular component (CC), and molecular function (MF) of potential targets were clustered based on the ClusterProfiler R package (version: 3.18.0). In the enrichment result, *p* < 0.05 or FDR < 0.05 were considered meaningful.

Using the “clusterProfiler” package in Bioconductor and the gene function spectrum obtained through enrichment analysis of GO and KEGG pathways, we found that DEGs were mainly concentrated in the following functional categories: Neutrophil extracellular trap formation, Lipid and atherosclerosis, IL−17 signaling pathway, Cytokine–cytokine receptor interaction, response to molecule of bacterial origin, neutrophil degranulation, neutrophil activation involved in immune response, defense response to bacterium (Figure 2).

### Identification and Validation of Biomarkers for Diagnostic Characteristics

**Figure 3:**
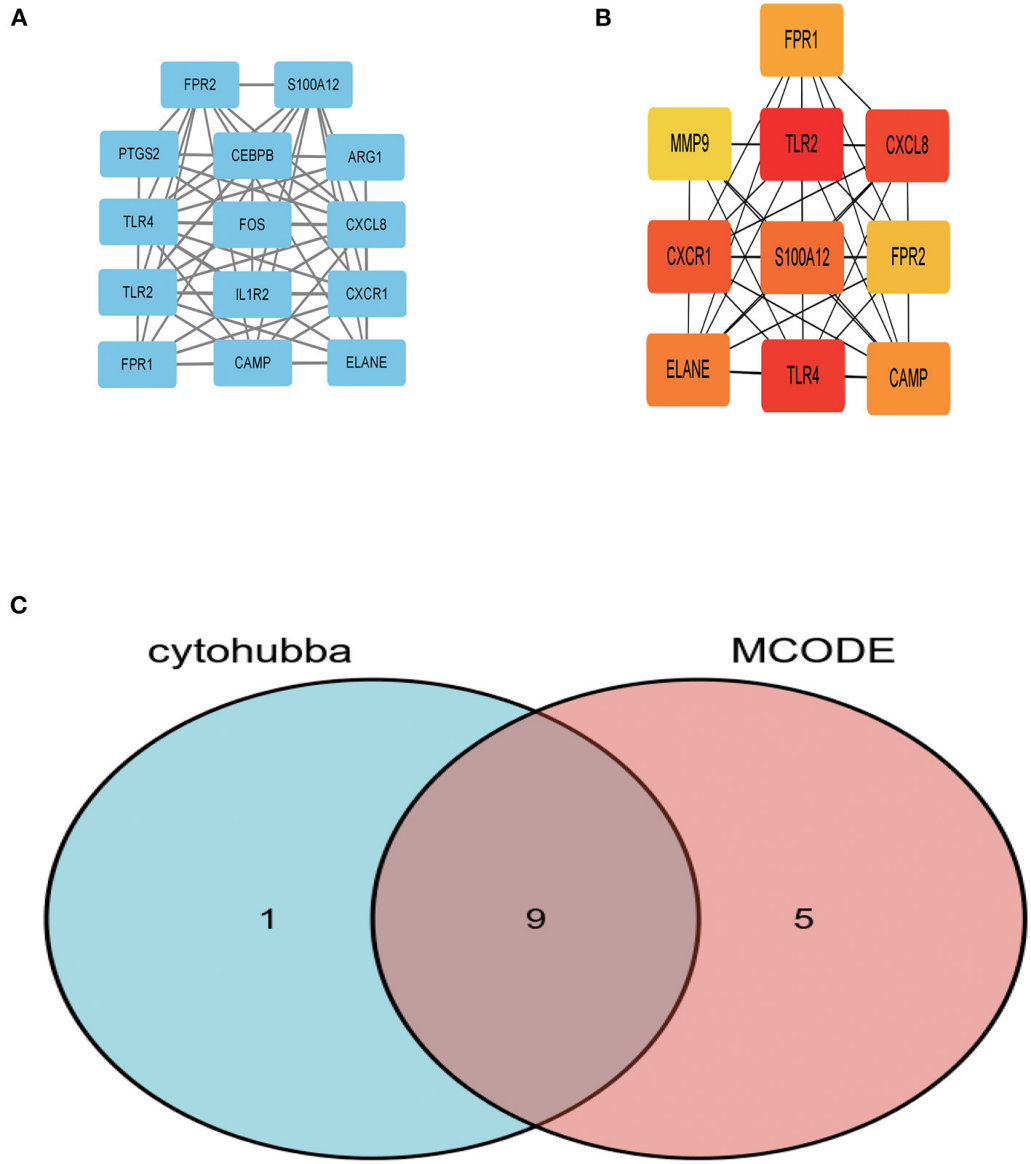
The key genes were identified using MCC and MCODE. **(A)** Represents the 10 key genes calculated by MCC algorithm in cytoHubba; the darker the color, the more critical the gene. **(B)** Denotes the gene related to the module with the highest score in MCODE calculation methods using Cytoscape. **(C)** The intersection of the key genes calculated by MCC and MCODE is visualized using Venn diagram.

In order to further explore central genes related to AMI and their mechanism of action, 157 genes with upregulated expression among the 210 DEGs in the AMI group were located and uploaded to STRING online database to build a PPI network. A PPI network with 156 genes as nodes and 85 edges was realized (Supplementary Material 1). Among the 156 nodes, the top 9 genes with high binding degree were found by Cytoscape (version 3.8.2) MCODE and MCC calculation methods. These genes, which were identified to play key roles in AMI, are listed as follows: *FPR1, CXCR1, ELANE, TLR2, S100A12, TLR4, CXCL8, FPR2* and *CAMP* (Figure 3).

### Diagnostic Effect of Characteristic Biomarkers on Acute Myocardial Infarction

**Figure 4:**
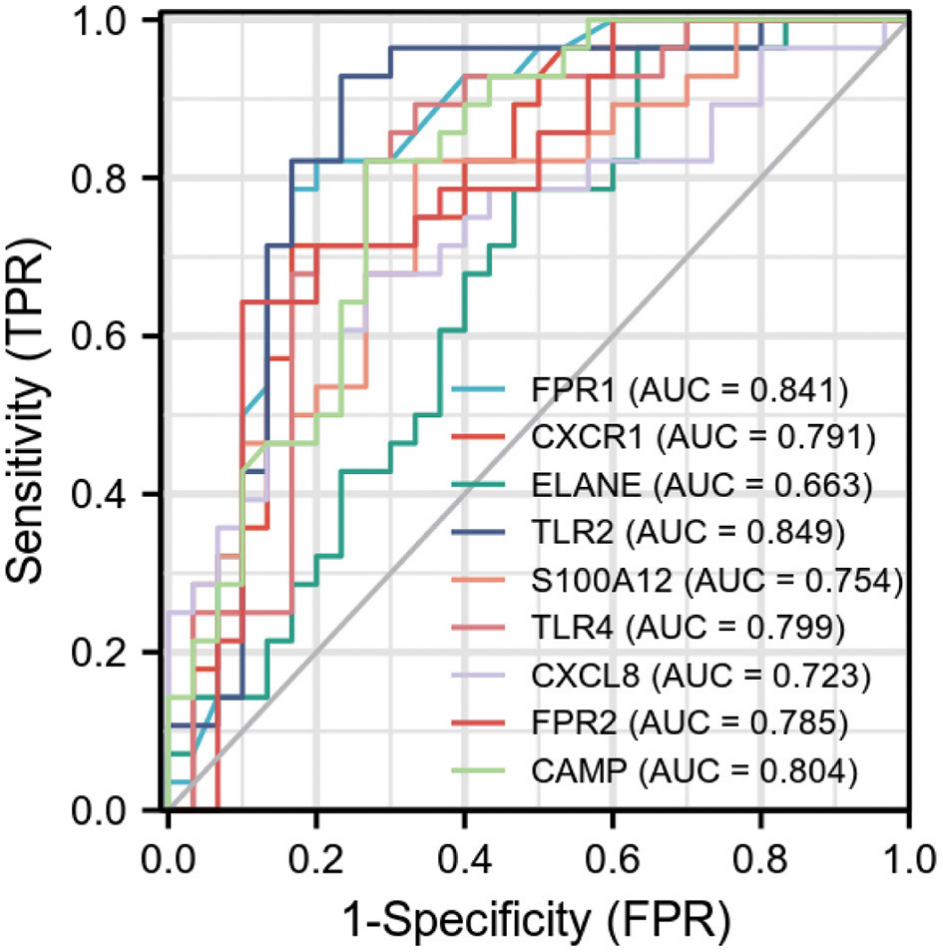
Receiver operating characteristic (ROC) curve of differentially expressed genes related to AMI, independence. TPR: true positive rate, the ratio of positive samples to all positive samples predicted by classifier, i.e., TP/(TP+FN); FPR: False negative rate, the ratio of positive classes to all negative classes in the sample predicted by the classifier, i.e., FP/(FP+TN). By changing different thresholds, a pair of TPR and FPR will be obtained. ROC curve is a curve drawn with FPR as abscissa and TPR as ordinate. As shown in the figure, each point on the curve corresponds to FPR and TPR at different thresholds. (The meaning of TPRate is the proportion of all samples of true category 1 that are predicted to be category 1. The meaning of FPRate is the proportion of all samples with true category 0 that are predicted to be category 1. AUC means that a positive sample and a negative sample are randomly selected from the sample. The probability that the classifier predicts the positive sample to be positive is P1, and the probability that the negative sample is positive is P2. AUC means the probability that P1 > P2).

Ten biomarkers were used to distinguish AMI from control samples demonstrating strongly predictive diagnostic results (Figure 4). The AUC value of *FPR1* was 0.841 (95% CI: 0.734–0.948), *CXCR1* was 0.791 (95% CI: 0.672–0.910), *ELANE* was 0.663 (95% CI: 0.522–0.804). The AUC value of *TLR2* was 0.849 (95% CI: 0.738–0.960), *S100A12* was 0.754 (95% CI: 0.627–0.880), and *TLR4* was 0.799 (95% CI: 0.678–0.919). The AUC value of *CXCL8* was 0.723 (95% CI: 0.589–0.856), *FPR2* was 0.785 (95% CI: 0.662–0.907), and *CAMP* was 0.804 (95% CI: 0.691–0.918).

### Acute Myocardial Infarction Is Associated With Immune Checkpoints, m6A, and Ferroptosis

**Figure 5:**
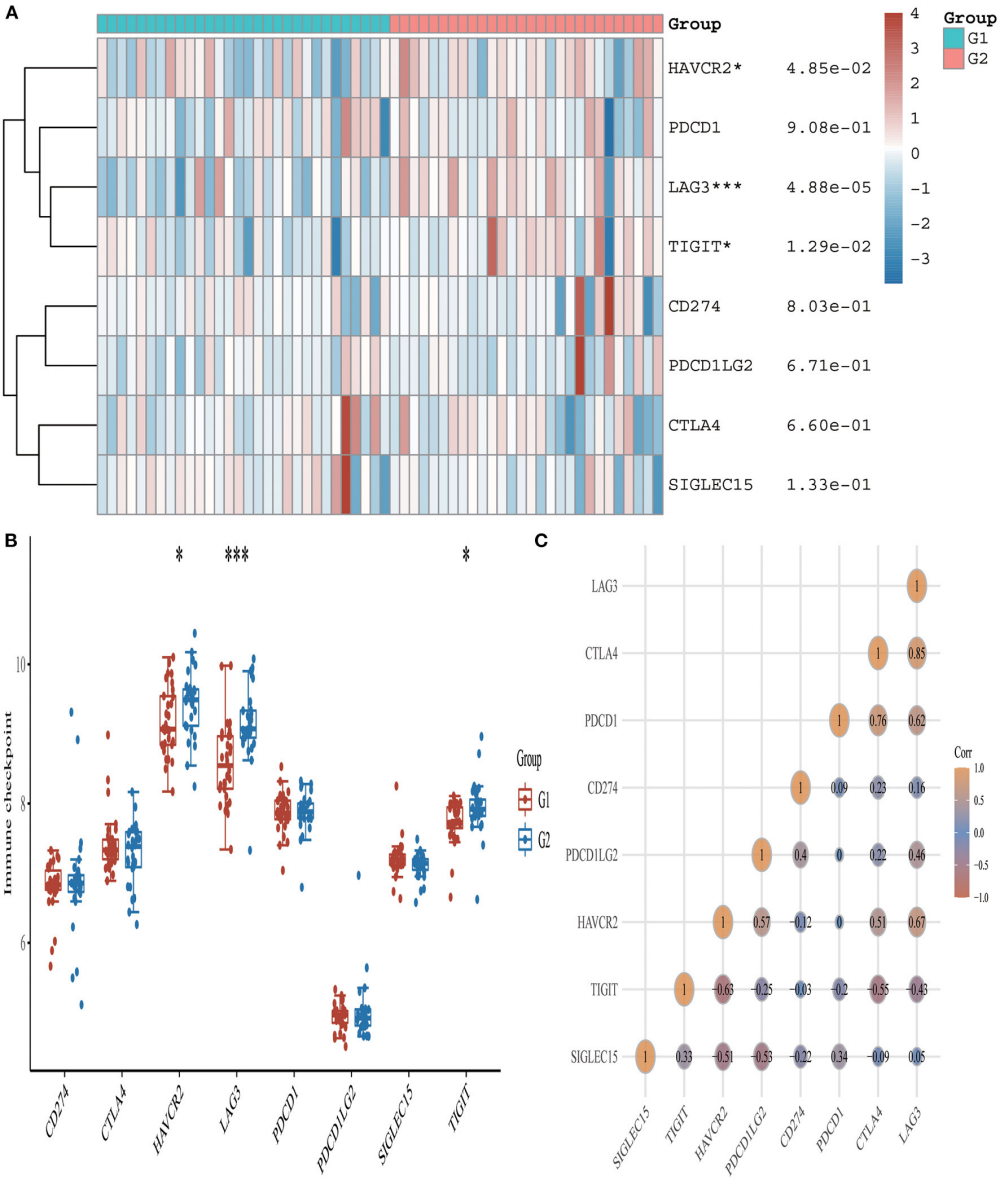
Expression pattern of immune checkpoint-related mRNAs in AMI and control groups. **(A)** Comparison of expression levels of 8 immune checkpoint-related RNA. Immune checkpoint-related RNA between healthy controls and AMI patients. G1 represents AMI patients and G2 represents healthy controls. *p < 0.05, ***p < 0.001, the asterisk represents the degree of importance (*p). **(B)** Visualization of differentially expressed regulators in AMI. The AMI patients were marked cyan, and the healthy controls were marked pink. *p < 0.05, **p < 0.01, ***p < 0.001, the asterisk represents the degree of importance (*p). **(C)** Spearman correlation analysis of 8 immune checkpoint-related RNA in AMI. The higher the number in the circle, the stronger the correlation. The change in color on the right represents a positive or negative correlation.

**Figure 6:**
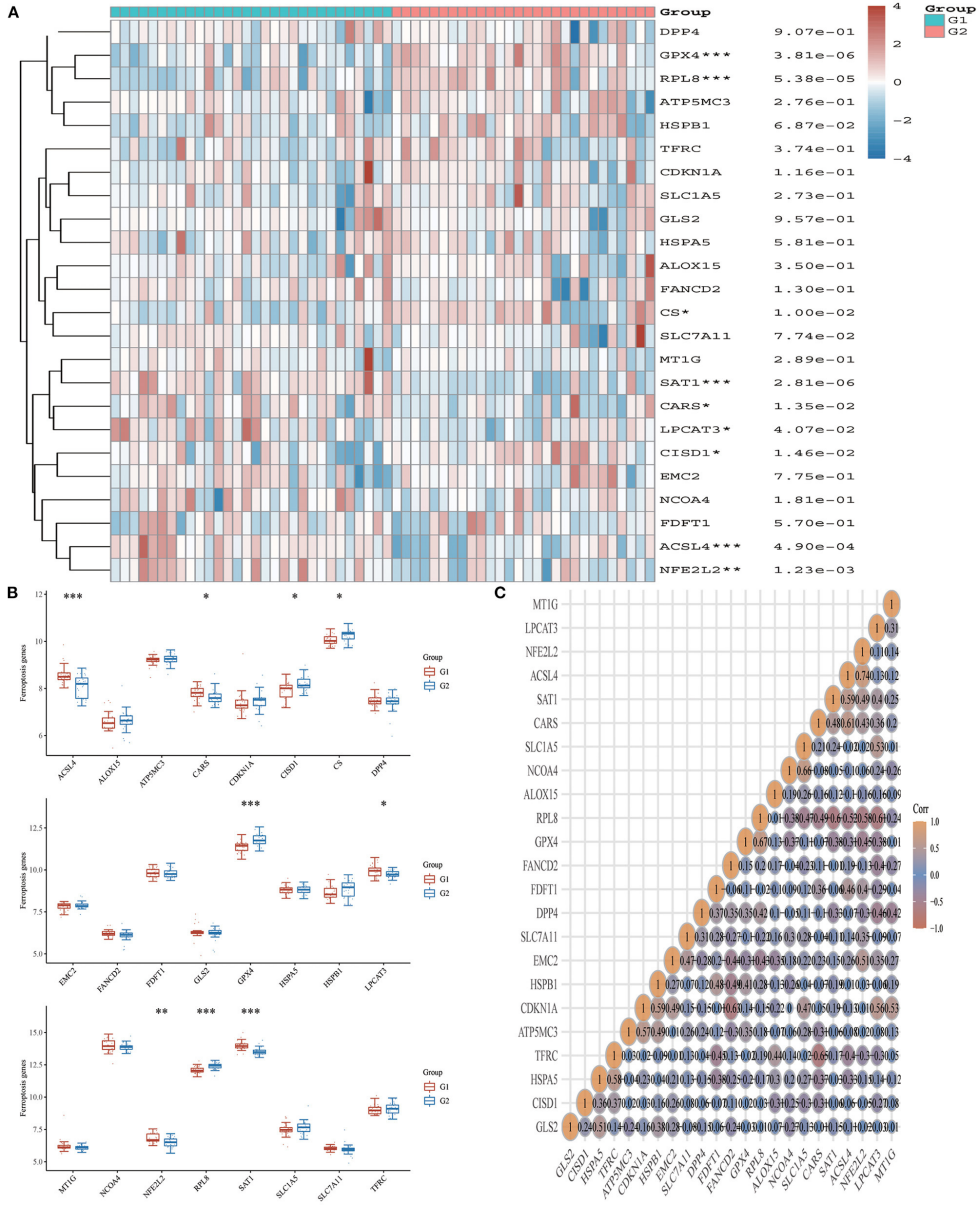
Expression pattern of ferroptosis-related mRNAs in AMI and control groups. **(A)** Comparison of expression levels of 24 ferroptosis-related RNA. Ferroptosis-related RNA between healthy controls and AMI patients. G1 represents AMI patients and G2 represents healthy controls. *p < 0.05, **p < 0.01, ***p < 0.001, the asterisk represents the degree of importance (*p). **(B)** Visualization of differentially expressed regulators in AMI. The AMI patients were marked cyan, and the healthy controls were marked pink. *p < 0.05, **p < 0.01, ***p < 0.001, the asterisk represents the degree of importance (*p). **(C)** Spearman correlation analysis of 24 ferroptosis-related RNA in AMI. The higher the number in the circle, the stronger the correlation. The change in color on the right represents a positive or negative correlation.

**Figure 7:**
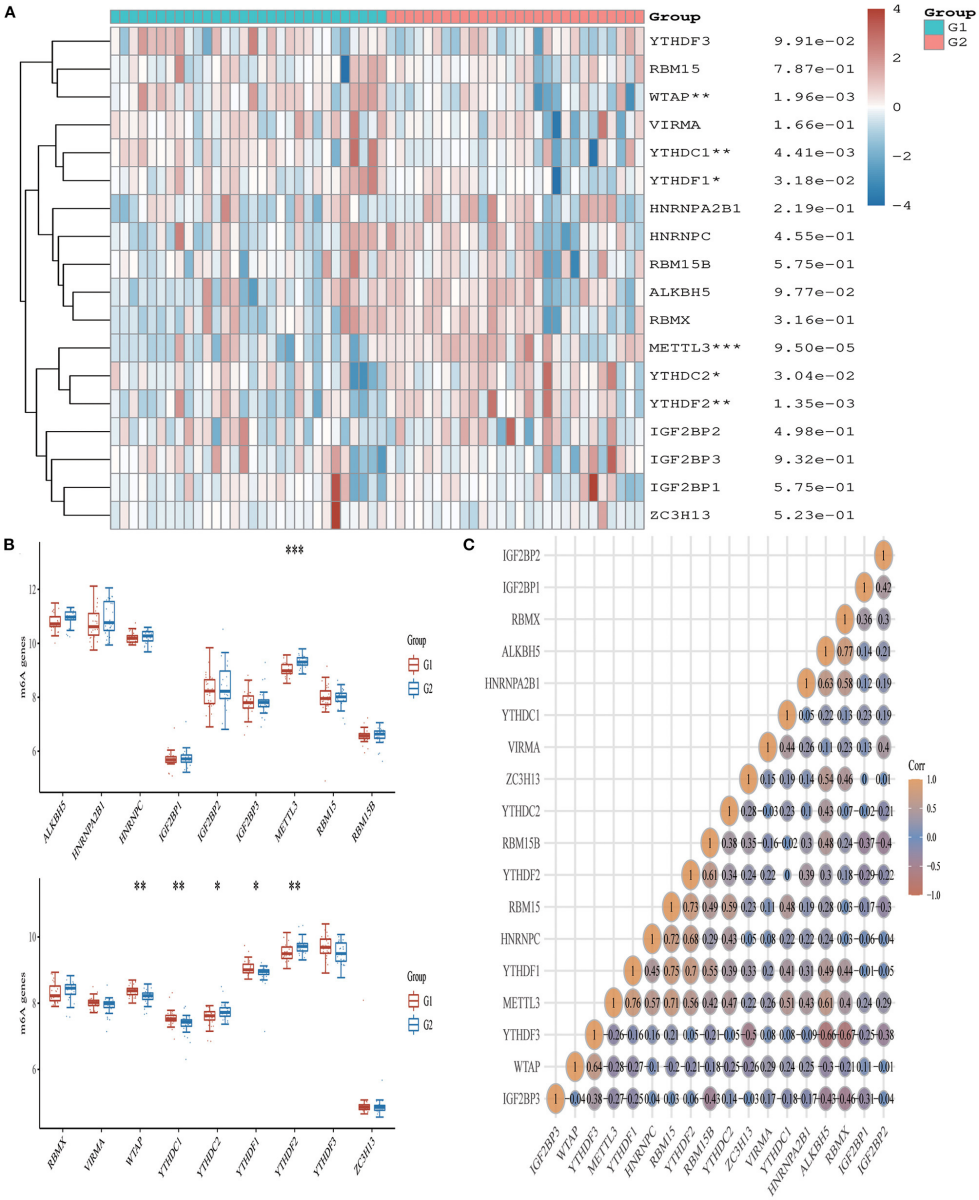
Expression pattern of m6A RNA methylation regulators in AMI. **(A)** Comparison of expression levels of 18 m6A RNA methylation regulators between AMI patients and healthy controls. *p < 0.05, **p < 0.01, ***p < 0.001, the asterisk represents the degree of importance (*p). **(B)** Visualization of differentially expressed regulators in AMI. The AMI patients were marked cyan, and the healthy controls were marked pink. *p < 0.05, **p < 0.01, ***p < 0.001, the asterisk represents the degree of importance (*p). **(C)** Spearman correlation analysis of 18 m6A-related RNA in AMI. The higher the number in the circle, the stronger the correlation. The change in color on the right represents a positive or negative correlation.

From the 24 ferroptosis-associated genes that were collected, changes were observed in gene expressions between patients with AMI and healthy controls. Among the patients, we observed that the ferroptosis-related genes *ACSL4, CARS, LPCAT3, NFE2L2* and *SAT1* were closely associated with AMI. Analysis of these ferroptosis-related genes showed that the strongest association was *ACSL4* and *NFE2L2, GPX4* and *PRL8*. Additionally, *CISD1, CS, CPX4* and *PRL8* expression in AMI were significantly lower than those in healthy controls. Studies have shown that high levels of the antioxidant enzyme glutathione peroxidase (GPx) are associated with improved prognosis after acute coronary syndrome (ACS) and have a protective effect (42, 45). Many regulators are involved in RNA methylation, including methyltransferase (Writer), RNA-binding protein (Reader), and demethylase (Erasers) (43, 44, 46). Therefore, we collected genes associated with these three regulatory types and investigated their association with AMI. We found that the expressions of *WTAP, YTHDC1* and *YTHDF1* were significantly increased in patients with AMI (P < 0.01). Analysis of these m6A related genes showed that the strongest association was *RBMX* and *ALKBH5, METTL3* and *YTHDF1*. However, *METTL3, YTHDC2* and *YTHDF2* in AMI were lower than those in healthy controls. During the verification of immune checkpoint, the expression level of *LAG3, HAVCR2*, and *TIGIT* were all lower than those of the healthy group (*P* < 0.01). Analysis of these immune checkpoint of AMI genes showed that the strongest association was *LAG3* and *CTLA4, CTLA4* and *PDCD1* (Figures 5–7).

### Validation of Diagnostic Genes

**Figure 8:**
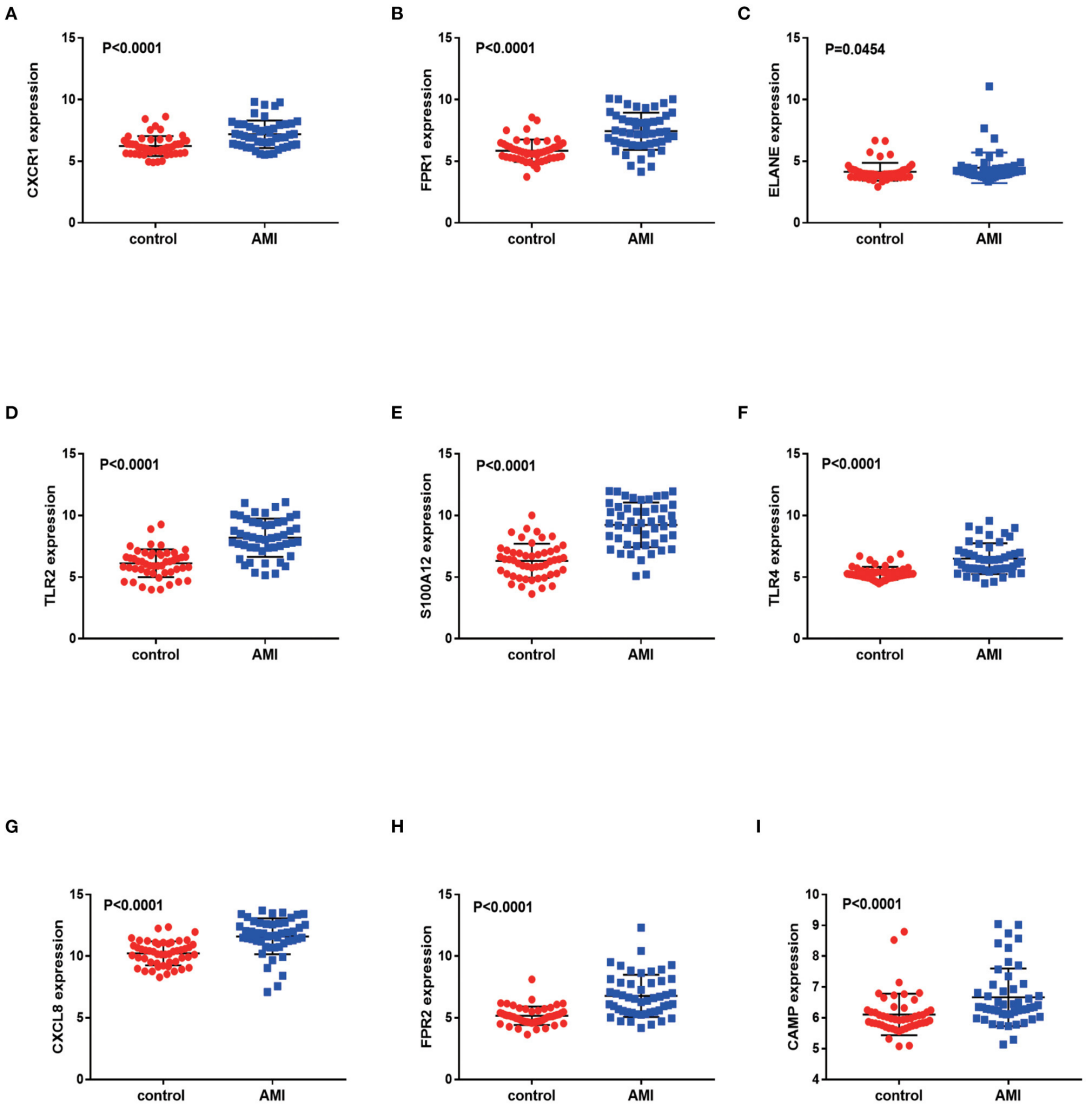
Validation of differentially expressed genes in AMI. The figure **(A–I)** shows the expression of differentially expressed genes in the GSE66360 data sets in AMI and non-AMI patients. The blue square represents gene expression in the AMI group, and the red circle represents gene expression in the healthy control group.

We validated DEGs from a new AMI-related dataset, GSE66360. Through verification, it was found that the *p-*values corresponding to *FPR1, CXCR1, ELANE, TLR2, S100A12, TLR4, CXCL8, FPR2* and *CAMP* are all less than 0.05. And the verification results partly support our conclusion that *FPR1, CXCR1, ELANE, TLR2, S100A12, TLR4, CXCL8, FPR2* and *CAMP* have the potential to be a marker for early diagnosis of AMI (Figure 8). We also validated ferroptosis, immune checkpoints, and m6A-related gene expression and found that the *p-*values corresponding to *NFE2L2, SAT1, WTAP* and *YTHDC1* are less than 0.05 (Supplementary Material 2).

## Discussion

Myocardial infarction is a leading cause of morbidity and mortality worldwide. Studies show that in 2015 alone, 15.9 million patients suffered from AMI (47). Despite significant improvements in the early diagnosis and treatment of AMI in the past decade, it remains a leading cause of death and disability. Therefore, the identification of new biomarkers for the early diagnosis of AMI requires further investigation. In recent years, RNA has emerged as a particular primary biomarker for cardiovascular disease. With the development of gene chip technology, microarrays have been widely used in heart disease research (48, 49). In this study, we first used the GEO gene expression dataset to detect differential gene expression associated with AMI and identify DEGs *via* functional analysis. Thereafter, we used MCC and MCODE to screen nine genes as potential diagnostic markers. We also analyzed the independent prediction ROC curve. Subsequently, we validated the association of AMI with immune checkpoints, ferroptosis, and m6A.

Comparing the expression levels of patient target genes that early predict STEMI development, we found significant differences in *FPR1, CXCR1, ELANE, TLR2, S100A12, TLR4, CXCL8, FPR2* and *CAMP* expressions, which significantly increased after STEMI. *CXCR1* is internally expressed on neutrophils and is responsible for chemotaxis and activation of neutrophils (50). *TLR* is a type I membrane binding protein that can recognize and defense against invading microorganisms (51). So far, it has been reported that *TLR2* induces a proinflammatory responses within immune cells. *S100A12* play an important role in the development of atherosclerosis (52). In cardiovascular diseases, *TLR4* has protective and harmful effects on some systems, such as mediating various inflammatory effects of the aorta (53), arterioles (54), fat cells and macrophages (55). *CXCL8* mainly arises from neutrophils, macrophages, monocytes, endothelial cells, epithelial cells, and T-cells (56). *FPR2* can inhibit inflammatory processes by inhibiting neutrophils, exerting anti-inflammatory and decomposition promoting properties (57). *CAMP* induces immune cells to the site of injury or infection to bind and neutralize lipopolysaccharide (LPS), thereby promoting epithelialization and repair the injury (58). Through the observation of these key genes, we found that these genes share common functional attributes, namely inflammation and immune response. Therefore, we consider that the future research direction of AMI still tends to attack inflammation and immunity, but of course, further basic experiments are still needed to verify.

Through functional verification of DEGs, it was found that the GO and KEGG pathways were mainly distributed in inflammation, immunity, and bacterial defense mechanisms. The occurrence and development of myocardial infarction involves almost all kinds of immune cells, some of which can lead to atherosclerosis and myocardial infarction, while others may prevent the corresponding lesions. Immune activation is the only way and early phenomenon of immune response. Previous studies have shown that the process of myocardial infarction is usually accompanied by the activation of host immune cells and the occurrence of inflammatory response, but it is not clear which immune cells are activated during the occurrence and progression of myocardial infarction. In GO and KEGG analysis of DEGs in patients with AMI, we found that a variety of immune and inflammatory cells, especially neutrophil, macrophage, and leukocyte, were widely activated in patients with AMI. Therefore, we believe that immune dysregulation or inflammation is significantly associated with AMI.

In recent years, ferroptosis has been a hot topic in investigations of atherosclerotic lesions, and frequent and long-term whole blood donation can reduce iron content in the body, which may be related to the reduced risk of atherosclerotic cardiovascular events (59). In our study on the relationship between DEGs and ferroptosis in AMI, we found that *NFE2L2* and *SAT1* genes related to ferroptosis were closely related to the occurrence of AMI. Similarly, in the analysis of m6A-related genes in patients with AMI, we found that methylation of *WTAP* and *YTHDC1* was closely related to the occurrence of AMI. Although we found no correlation between immune checkpoint and the occurrence and development of AMI in gene expression, we found reliable immune checkpoint interaction in AMI patients in the relationship between immune checkpoint gene interaction. In conclusion, we suspect that the occurrence of AMI is related to the immune checkpoint, ferroptosis, and m6A. Nevertheless, at present, we have only detected different expressions of genes related to immune checkpoint, ferroptosis or m6A, and we have not further explored how to play a role in the occurrence and development of AMI. Therefore, this path can be further explored in the future. But this hypothesis needs to be verified.

This study has some limitations. For example, there are several studies on the differential expression of AMI genes. However, the results of those studies are different to this study. This could be due to the following reasons: (1) different batches of microarray analyses have different results to some extent; (2) compared with other studies, this study adopted three AMI data sets, providing a comprehensive analysis method for bioinformatics for AMI. Therefore, the results of this study are reliable. In addition, the reproducibility of immune checkpoint-, ferroptosis-, and m6A-related genes obtained from the dataset needs to be further validated. Further large-scale basic studies can be carried out to verify the conclusions of this study.

The timely diagnosis and treatment of AMI can help improve global health. Considering this, our study aimed to identify new genetic markers associated with AMI. We found nine genes related to the occurrence of AMI. Furthermore, we believe that the occurrence of AMI is related to immune checkpoint, ferroptosis, and m6A.

## Data Availability Statement

The raw data supporting the conclusions of this article will be made available by the authors, without undue reservation.

## Author Contributions

XT conceived and designed the study, conducted the experiments, analyzed the data, interpreted the results, and drafted the manuscript. XZ and XD prepared the charts. YK and JK edited it. All authors contributed to the article and approved the submitted version.

## Funding

This study was supported by the National Natural Science Foundation project of China (Grant Number: 81970419).

## Conflict of Interest

The authors declare that the research was conducted in the absence of any commercial or financial relationships that could be construed as a potential conflict of interest.

## Publisher's Note

All claims expressed in this article are solely those of the authors and do not necessarily represent those of their affiliated organizations, or those of the publisher, the editors and the reviewers. Any product that may be evaluated in this article, or claim that may be made by its manufacturer, is not guaranteed or endorsed by the publisher.

## Footnotes

^1^http://www.ncbi.nih.gov/geo.
