# Supplementary material for: Predicting Diagnostic Gene Biomarkers Associated With Immune Checkpoints, N6-Methyladenosine, and Ferroptosis in Patients With Acute Myocardial Infarction
Source: Front Cardiovasc Med. 2022 Feb 11;9:836067. doi: 10.3389/fcvm.2022.836067 (PMC8873927; doi:10.3389/fcvm.2022.836067)
Supplement: Supplementary file 1 [file Table_1.DOCX]

group1 group2 number Min Max Median IQR Mean SD SE

FPR1 group1 30 13.409 15.424 14.973 0.652 14.866 0.499 0.091

FPR1 group2 28 13.050 15.042 14.330 0.527 14.247 0.451 0.085

CXCR1 group1 30 9.561 13.399 12.049 0.788 11.995 0.718 0.131

CXCR1 group2 28 10.188 12.469 11.395 0.663 11.403 0.528 0.100

ELANE group1 30 6.477 11.123 8.288 1.613 8.461 1.219 0.223

ELANE group2 28 6.019 9.824 7.679 0.933 7.744 0.833 0.157

TLR2 group1 30 8.819 11.038 10.067 0.643 9.985 0.549 0.100

TLR2 group2 28 8.758 10.425 9.310 0.281 9.317 0.340 0.064

S100A12 group1 30 9.076 13.716 11.822 1.136 11.798 0.943 0.172

S100A12 group2 28 9.768 12.482 10.986 1.269 11.038 0.745 0.141

TLR4 group1 30 8.850 11.307 10.448 0.784 10.299 0.619 0.113

TLR4 group2 28 8.913 10.676 9.610 0.604 9.641 0.441 0.083

CXCL8 group1 30 7.521 13.388 9.386 1.364 9.442 1.291 0.236

CXCL8 group2 28 4.891 12.026 8.296 1.548 8.301 1.501 0.284

FPR2 group1 30 8.671 12.181 11.111 0.860 11.040 0.783 0.143

FPR2 group2 28 9.448 11.356 10.405 0.761 10.440 0.556 0.105

CAMP group1 30 9.896 13.535 11.680 1.203 11.723 0.969 0.177

CAMP group2 28 9.058 11.756 10.756 0.951 10.696 0.727 0.137
