# Supplementary material for: Predicting Diagnostic Gene Biomarkers Associated With Immune Checkpoints, N6-Methyladenosine, and Ferroptosis in Patients With Acute Myocardial Infarction
Source: Front Cardiovasc Med. 2022 Feb 11;9:836067. doi: 10.3389/fcvm.2022.836067 (PMC8873927; doi:10.3389/fcvm.2022.836067)
Supplement: Supplementary file 2 [file Table_2.DOCX]

CXCR1

| Table Analyzed | Unpaired t test data |
| --- | --- |
|  |  |
| Column B | AMI |
| vs. | vs. |
| Column A | **control** |
|  |  |
| Mann Whitney test |  |
| P value | <0.0001 |
| Exact or approximate P value? | Exact |
| P value summary | **** |
| Significantly different (P < 0.05)? | Yes |
| One- or two-tailed P value? | Two-tailed |
| Sum of ranks in column A,B | 1862 , 3088 |
| Mann-Whitney U | 587 |
|  |  |
| Difference between medians |  |
| Median of column A | 6.101, n=50 |
| Median of column B | 7.049, n=49 |
| Difference: Actual | 0.9479 |
| Difference: Hodges-Lehmann | 0.8838 |

FPR1

| Table Analyzed | Unpaired t test data |
| --- | --- |
|  |  |
| Column B | AMI |
| vs. | vs. |
| Column A | control |
|  |  |
| Mann Whitney test |  |
| P value | <0.0001 |
| Exact or approximate P value? | Exact |
| P value summary | **** |
| Significantly different (P < 0.05)? | Yes |
| One- or two-tailed P value? | Two-tailed |
| Sum of ranks in column A,B | 1716 , 3234 |
| Mann-Whitney U | 441 |
|  |  |
| Difference between medians |  |
| Median of column A | 5.703, n=50 |
| Median of column B | 7.352, n=49 |
| Difference: Actual | 1.65 |
| Difference: Hodges-Lehmann | 1.586 |

ELANE

| Table Analyzed | Unpaired t test data |
| --- | --- |
|  |  |
| Column B | AMI |
| vs. | vs. |
| Column A | control |
|  |  |
| Mann Whitney test |  |
| P value | 0.0454 |
| Exact or approximate P value? | Exact |
| P value summary | * |
| Significantly different (P < 0.05)? | Yes |
| One- or two-tailed P value? | Two-tailed |
| Sum of ranks in column A,B | 2214 , 2736 |
| Mann-Whitney U | 939 |
|  |  |
| Difference between medians |  |
| Median of column A | 3.955, n=50 |
| Median of column B | 4.2, n=49 |
| Difference: Actual | 0.2449 |
| Difference: Hodges-Lehmann | 0.1828 |

TLR2

| Table Analyzed | Unpaired t test data |
| --- | --- |
|  |  |
| Column B | AMI |
| vs. | vs. |
| Column A | control |
|  |  |
| Mann Whitney test |  |
| P value | <0.0001 |
| Exact or approximate P value? | Exact |
| P value summary | **** |
| Significantly different (P < 0.05)? | Yes |
| One- or two-tailed P value? | Two-tailed |
| Sum of ranks in column A,B | 1629 , 3321 |
| Mann-Whitney U | 354 |
|  |  |
| Difference between medians |  |
| Median of column A | 6.148, n=50 |
| Median of column B | 8.165, n=49 |
| Difference: Actual | 2.017 |
| Difference: Hodges-Lehmann | 2.104 |

S100A12

| Table Analyzed | Unpaired t test data |
| --- | --- |
|  |  |
| Column B | AMI |
| vs. | vs. |
| Column A | control |
|  |  |
| Mann Whitney test |  |
| P value | <0.0001 |
| Exact or approximate P value? | Exact |
| P value summary | **** |
| Significantly different (P < 0.05)? | Yes |
| One- or two-tailed P value? | Two-tailed |
| Sum of ranks in column A,B | 1534 , 3416 |
| Mann-Whitney U | 259 |
|  |  |
| Difference between medians |  |
| Median of column A | 6.382, n=50 |
| Median of column B | 9.359, n=49 |
| Difference: Actual | 2.977 |
| Difference: Hodges-Lehmann | 3.005 |

TLR4

| Table Analyzed | Unpaired t test data |
| --- | --- |
|  |  |
| Column B | AMI |
| vs. | vs. |
| Column A | control |
|  |  |
| Mann Whitney test |  |
| P value | <0.0001 |
| Exact or approximate P value? | Exact |
| P value summary | **** |
| Significantly different (P < 0.05)? | Yes |
| One- or two-tailed P value? | Two-tailed |
| Sum of ranks in column A,B | 1701 , 3249 |
| Mann-Whitney U | 426 |
|  |  |
| Difference between medians |  |
| Median of column A | 5.219, n=50 |
| Median of column B | 6.26, n=49 |
| Difference: Actual | 1.041 |
| Difference: Hodges-Lehmann | 0.9669 |

CXCL8

| Table Analyzed | Unpaired t test data |
| --- | --- |
|  |  |
| Column B | AMI |
| vs. | vs. |
| Column A | **control** |
|  |  |
| Mann Whitney test |  |
| P value | <0.0001 |
| Exact or approximate P value? | Exact |
| P value summary | **** |
| Significantly different (P < 0.05)? | Yes |
| One- or two-tailed P value? | Two-tailed |
| Sum of ranks in column A,B | 1708 , 3242 |
| Mann-Whitney U | 433 |
|  |  |
| Difference between medians |  |
| Median of column A | 10.28, n=50 |
| Median of column B | 11.68, n=49 |
| Difference: Actual | 1.407 |
| Difference: Hodges-Lehmann | 1.51 |

FPR2

| Table Analyzed | Unpaired t test data |
| --- | --- |
|  |  |
| Column B | AMI |
| vs. | vs. |
| Column A | control |
|  |  |
| Mann Whitney test |  |
| P value | <0.0001 |
| Exact or approximate P value? | Exact |
| P value summary | **** |
| Significantly different (P < 0.05)? | Yes |
| One- or two-tailed P value? | Two-tailed |
| Sum of ranks in column A,B | 1720 , 3230 |
| Mann-Whitney U | 445 |
|  |  |
| Difference between medians |  |
| Median of column A | 5.11, n=50 |
| Median of column B | 6.546, n=49 |
| Difference: Actual | 1.437 |
| Difference: Hodges-Lehmann | 1.36 |

CAMP

| Table Analyzed | Unpaired t test data |
| --- | --- |
|  |  |
| Column B | AMI |
| vs. | vs. |
| Column A | control |
|  |  |
| Mann Whitney test |  |
| P value | <0.0001 |
| Exact or approximate P value? | Exact |
| P value summary | **** |
| Significantly different (P < 0.05)? | Yes |
| One- or two-tailed P value? | Two-tailed |
| Sum of ranks in column A,B | 1923 , 3027 |
| Mann-Whitney U | 648 |
|  |  |
| Difference between medians |  |
| Median of column A | 5.96, n=50 |
| Median of column B | 6.352, n=49 |
| Difference: Actual | 0.3918 |
| Difference: Hodges-Lehmann | 0.4296 |
