# Supplementary material for: Predicting Diagnostic Gene Biomarkers Associated With Immune Checkpoints, N6-Methyladenosine, and Ferroptosis in Patients With Acute Myocardial Infarction
Source: Front Cardiovasc Med. 2022 Feb 11;9:836067. doi: 10.3389/fcvm.2022.836067 (PMC8873927; doi:10.3389/fcvm.2022.836067)
Supplement: Supplementary file 3 [file Data_Sheet_1.ZIP › supplementary materials/supplementary materials raw data/supplementary materials 2 raw data.docx]

ACSL4

| Table Analyzed | Unpaired t test data |
| --- | --- |
|  |  |
| Column B | AMI |
| vs. | vs. |
| Column A | **control** |
|  |  |
| Mann Whitney test |  |
| P value | 0.1069 |
| Exact or approximate P value? | Exact |
| P value summary | ns |
| Significantly different (P < 0.05)? | No |
| One- or two-tailed P value? | Two-tailed |
| Sum of ranks in column A,B | 2269 , 2681 |
| Mann-Whitney U | 994 |
|  |  |
| Difference between medians |  |
| Median of column A | 7.031, n=50 |
| Median of column B | 7.299, n=49 |
| Difference: Actual | 0.2685 |
| Difference: Hodges-Lehmann | 0.3029 |

CARS

| Table Analyzed | Unpaired t test data |
| --- | --- |
|  |  |
| Column B | AMI |
| vs. | vs. |
| Column A | **control** |
|  |  |
| Mann Whitney test |  |
| P value | 0.2423 |
| Exact or approximate P value? | Exact |
| P value summary | ns |
| Significantly different (P < 0.05)? | No |
| One- or two-tailed P value? | Two-tailed |
| Sum of ranks in column A,B | 2668 , 2282 |
| Mann-Whitney U | 1057 |
|  |  |
| Difference between medians |  |
| Median of column A | 6.973, n=50 |
| Median of column B | 6.957, n=49 |
| Difference: Actual | -0.0162 |
| Difference: Hodges-Lehmann | -0.1116 |

LPCAT3

| Table Analyzed | Unpaired t test data |
| --- | --- |
|  |  |
| Column B | AMI |
| vs. | vs. |
| Column A | control |
|  |  |
| Mann Whitney test |  |
| P value | 0.2472 |
| Exact or approximate P value? | Exact |
| P value summary | ns |
| Significantly different (P < 0.05)? | No |
| One- or two-tailed P value? | Two-tailed |
| Sum of ranks in column A,B | 2334 , 2616 |
| Mann-Whitney U | 1059 |
|  |  |
| Difference between medians |  |
| Median of column A | 7.443, n=50 |
| Median of column B | 7.561, n=49 |
| Difference: Actual | 0.118 |
| Difference: Hodges-Lehmann | 0.1087 |

NFE2L2

| Table Analyzed | Unpaired t test data |
| --- | --- |
|  |  |
| Column B | AMI |
| vs. | vs. |
| Column A | control |
|  |  |
| Mann Whitney test |  |
| P value | <0.0001 |
| Exact or approximate P value? | Exact |
| P value summary | **** |
| Significantly different (P < 0.05)? | Yes |
| One- or two-tailed P value? | Two-tailed |
| Sum of ranks in column A,B | 1768 , 3182 |
| Mann-Whitney U | 493 |
|  |  |
| Difference between medians |  |
| Median of column A | 6.392, n=50 |
| Median of column B | 6.979, n=49 |
| Difference: Actual | 0.5869 |
| Difference: Hodges-Lehmann | 0.6071 |

SAT1

| Table Analyzed | Unpaired t test data |
| --- | --- |
|  |  |
| Column B | AMI |
| vs. | vs. |
| Column A | control |
|  |  |
| Mann Whitney test |  |
| P value | <0.0001 |
| Exact or approximate P value? | Exact |
| P value summary | **** |
| Significantly different (P < 0.05)? | Yes |
| One- or two-tailed P value? | Two-tailed |
| Sum of ranks in column A,B | 1684 , 3266 |
| Mann-Whitney U | 409 |
|  |  |
| Difference between medians |  |
| Median of column A | 9.17, n=50 |
| Median of column B | 10.07, n=49 |
| Difference: Actual | 0.8967 |
| Difference: Hodges-Lehmann | 0.8399 |

WTAP

| Table Analyzed | Unpaired t test data |
| --- | --- |
|  |  |
| Column B | AMI |
| vs. | vs. |
| Column A | control |
|  |  |
| Mann Whitney test |  |
| P value | 0.0001 |
| Exact or approximate P value? | Exact |
| P value summary | *** |
| Significantly different (P < 0.05)? | Yes |
| One- or two-tailed P value? | Two-tailed |
| Sum of ranks in column A,B | 1964 , 2986 |
| Mann-Whitney U | 689 |
|  |  |
| Difference between medians |  |
| Median of column A | 8.194, n=50 |
| Median of column B | 8.661, n=49 |
| Difference: Actual | 0.4664 |
| Difference: Hodges-Lehmann | 0.3907 |

YTHDC1

| Table Analyzed | Unpaired t test data |
| --- | --- |
|  |  |
| Column B | AMI |
| vs. | vs. |
| Column A | control |
|  |  |
| Mann Whitney test |  |
| P value | 0.0011 |
| Exact or approximate P value? | Exact |
| P value summary | ** |
| Significantly different (P < 0.05)? | Yes |
| One- or two-tailed P value? | Two-tailed |
| Sum of ranks in column A,B | 2038 , 2912 |
| Mann-Whitney U | 763 |
|  |  |
| Difference between medians |  |
| Median of column A | 8.366, n=50 |
| Median of column B | 8.693, n=49 |
| Difference: Actual | 0.3273 |
| Difference: Hodges-Lehmann | 0.2991 |

YTHDF1

| Table Analyzed | Unpaired t test data |
| --- | --- |
|  |  |
| Column B | AMI |
| vs. | vs. |
| Column A | control |
|  |  |
| Mann Whitney test |  |
| P value | 0.2678 |
| Exact or approximate P value? | Exact |
| P value summary | ns |
| Significantly different (P < 0.05)? | No |
| One- or two-tailed P value? | Two-tailed |
| Sum of ranks in column A,B | 2659 , 2291 |
| Mann-Whitney U | 1066 |
|  |  |
| Difference between medians |  |
| Median of column A | 12.37, n=50 |
| Median of column B | 12.32, n=49 |
| Difference: Actual | -0.04506 |
| Difference: Hodges-Lehmann | -0.06483 |
