# Supplementary figures and images for: Predicting Diagnostic Gene Biomarkers Associated With Immune Checkpoints, N6-Methyladenosine, and Ferroptosis in Patients With Acute Myocardial Infarction
Source: Front Cardiovasc Med. 2022 Feb 11;9:836067. doi: 10.3389/fcvm.2022.836067 (PMC8873927; doi:10.3389/fcvm.2022.836067)

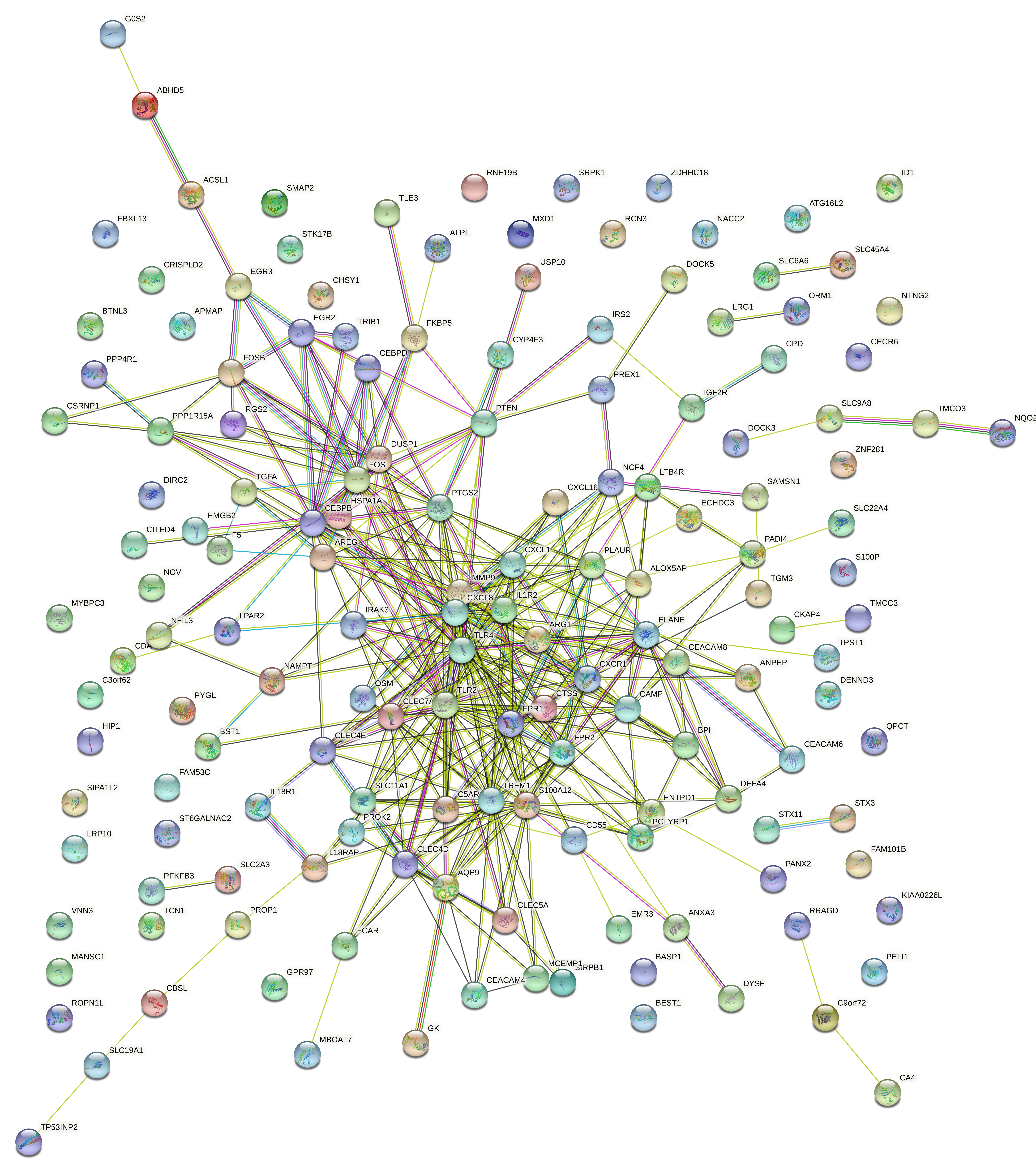

Supplement: Supplementary file 3 [file Data_Sheet_1.ZIP › supplementary materials/supplementary materials 1 .png]

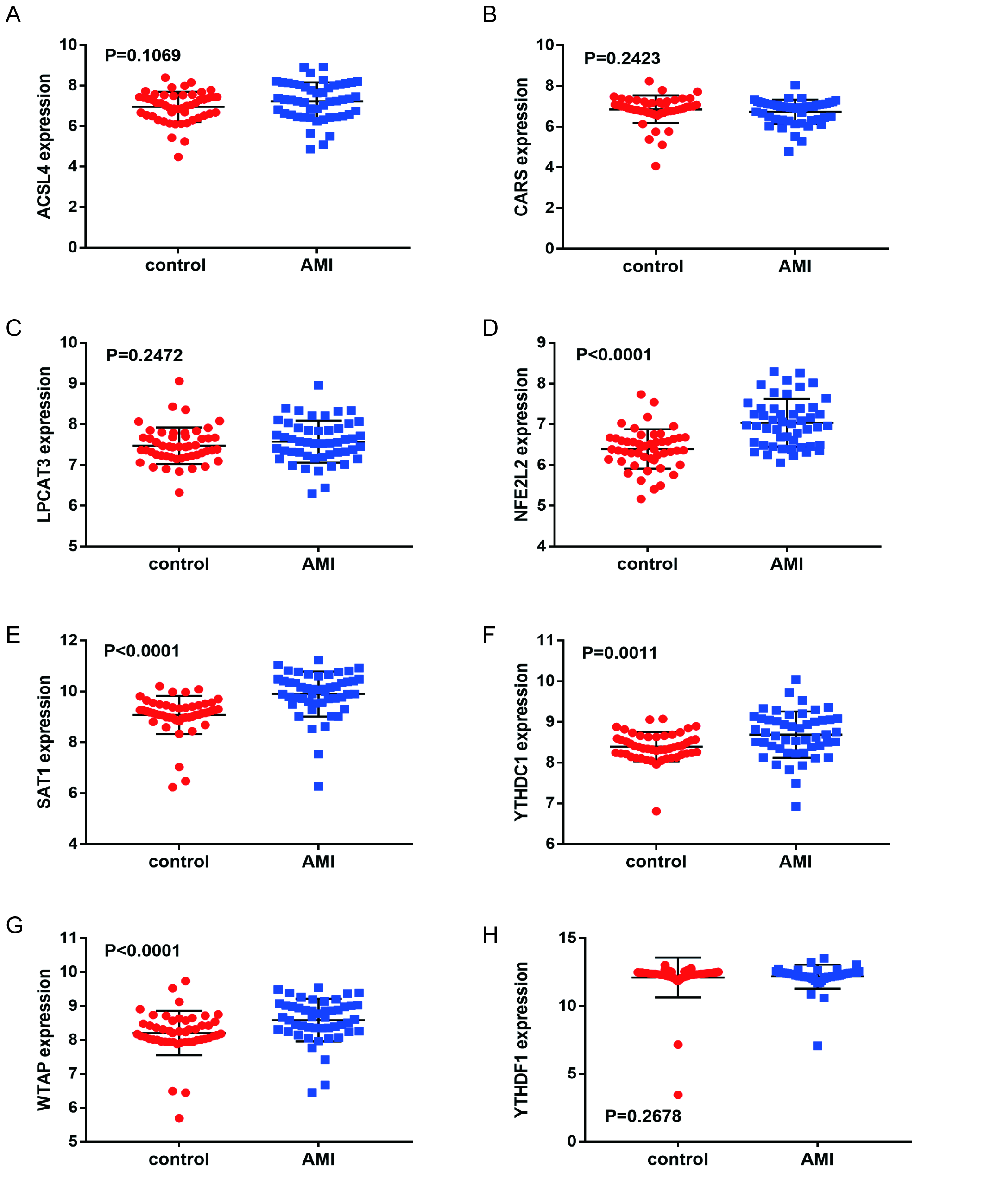

Supplement: Supplementary file 3 [file Data_Sheet_1.ZIP › supplementary materials/supplementary materials 2.tif]

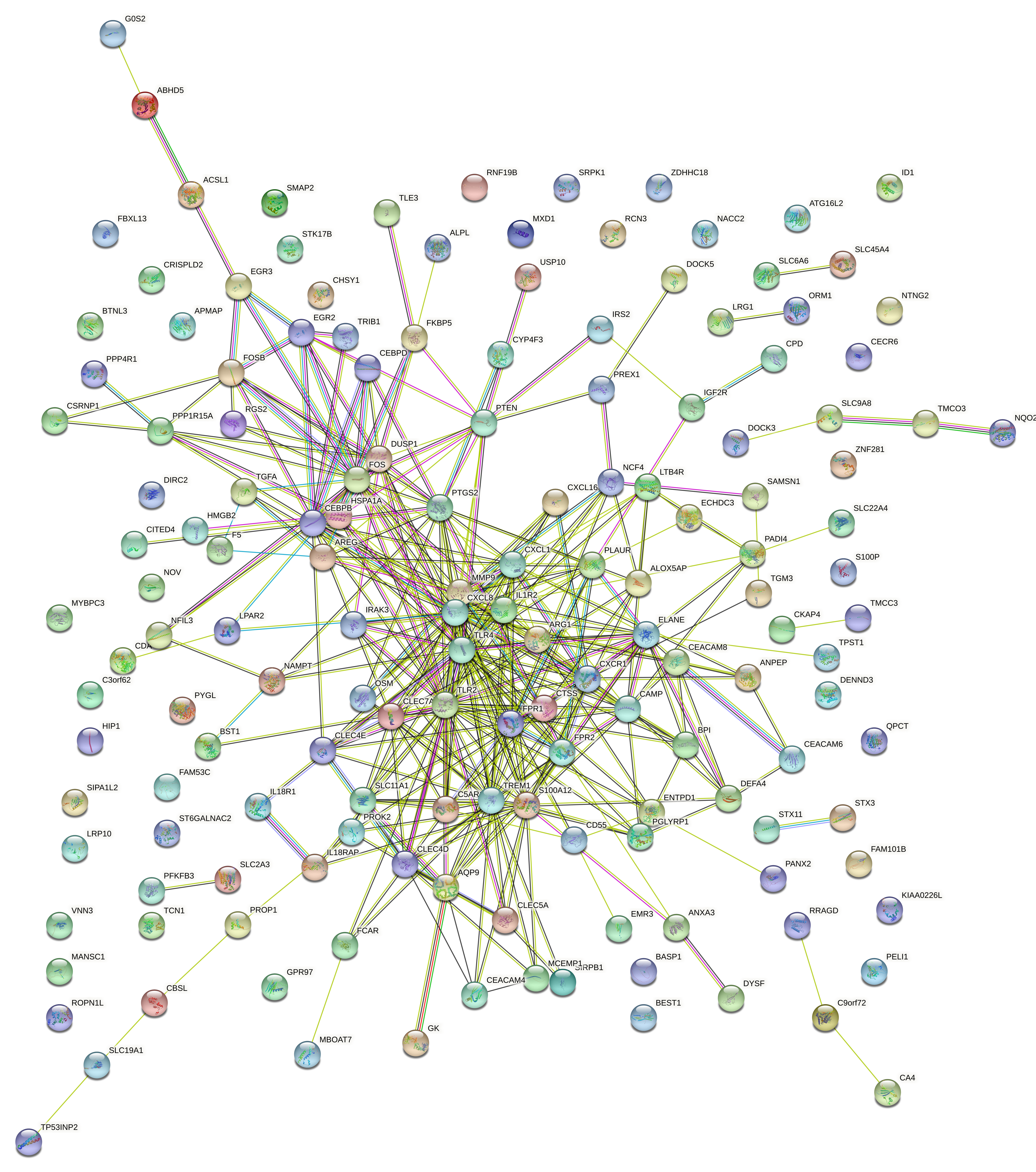

Supplement: Supplementary file 11 [file Data_Sheet_9.zip › supplementary materials pictures/supplementary materials 1 .png]
