# Supplementary material for: Predicting Diagnostic Gene Biomarkers Associated With Immune Checkpoints, N6-Methyladenosine, and Ferroptosis in Patients With Acute Myocardial Infarction
Source: Front Cardiovasc Med. 2022 Feb 11;9:836067. doi: 10.3389/fcvm.2022.836067 (PMC8873927; doi:10.3389/fcvm.2022.836067)
Supplement: Supplementary file 12 [file Data_Sheet_10.zip › supplementary materials pictures/Supplementary_Material.docx]

Supplementary Material


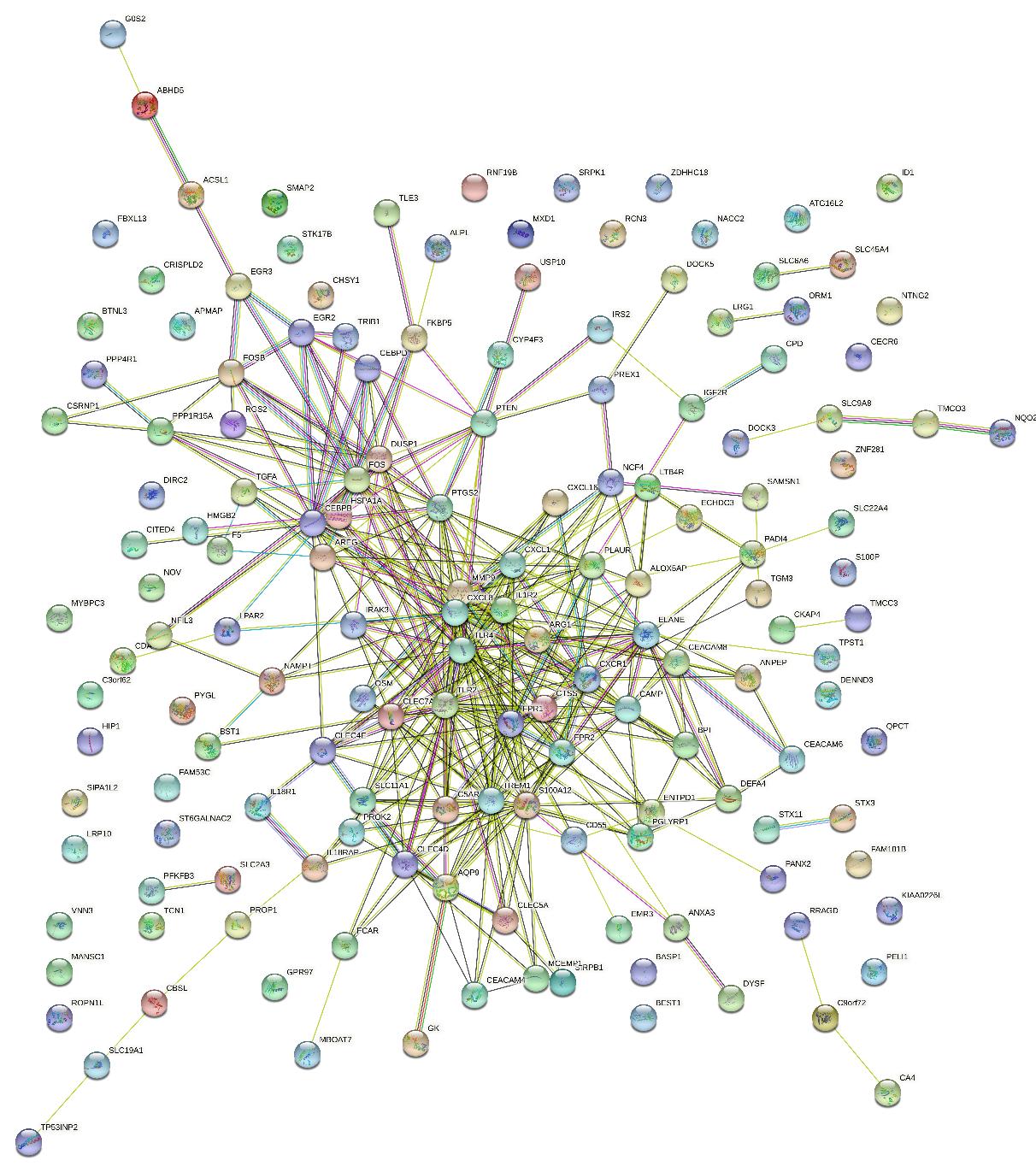


**Supplementary Figure 1. Display of protein interactions through the STRING database**

In the PPI network, nodes with fillers inside indicate that the 3D structure of the protein was known or predicted, and empty nodes indicate that the 3D structure was unknown. The connections between proteins represent the predicted functional associations that are specific and meaningful. There are seven differently-colored lines: 1. Light blue for database auxiliary evidence; 2. Purple for experimental proof; 3. Red for gene fusion; 4. Yellow-green for evidence mined from the article; 5. Green for gene close; 6. Blue for gene co-generation; and 7. Black for gene co-expression.


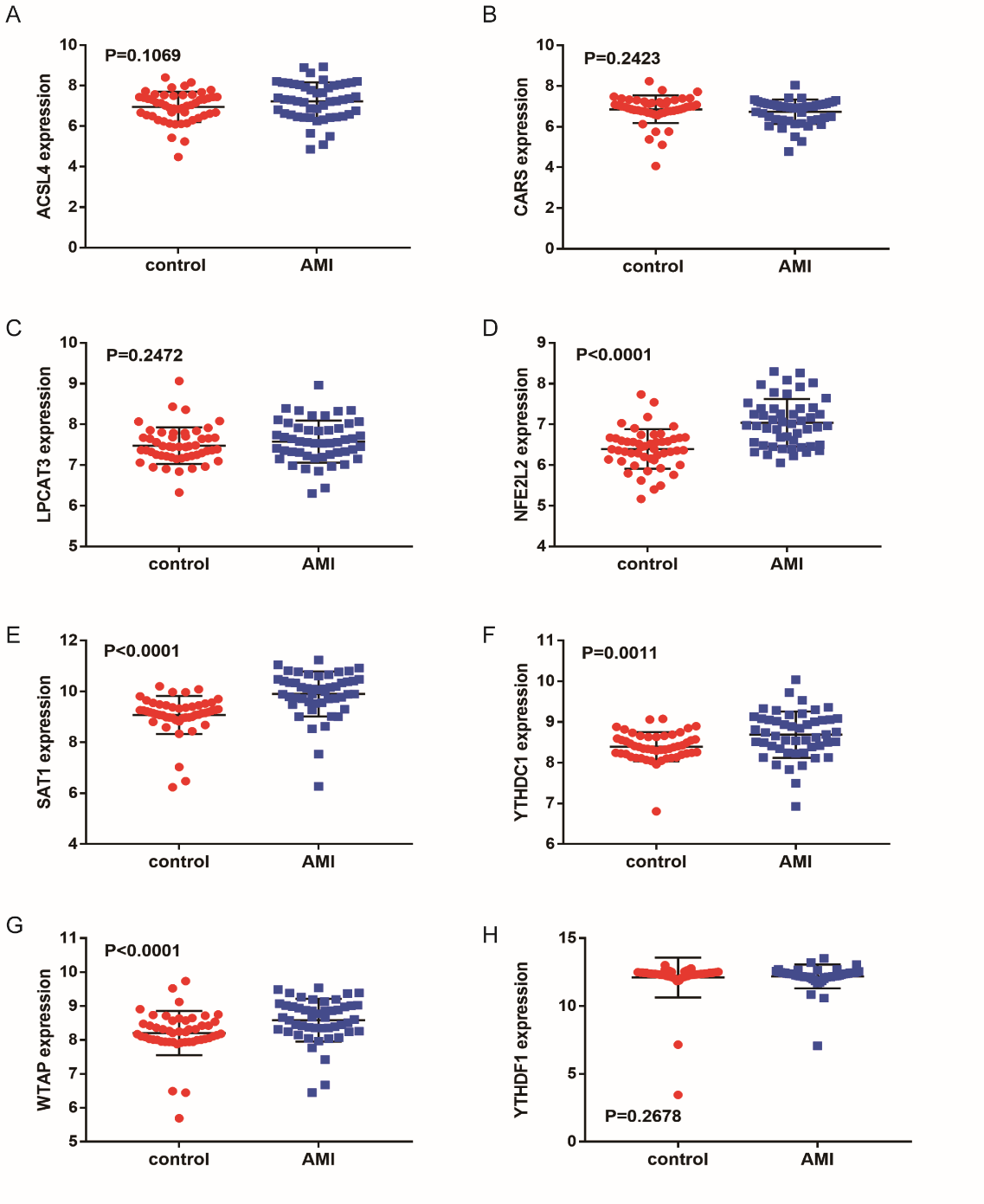


**Supplementary Figure 2. Validation of** **the immune checkpoint-, m6A-, and ferroptosis-related gene expression in AMI**

The A-H figures show the expression of the immune checkpoint-, m6A-, and ferroptosis-related genes in the GSE66360 data sets in AMI and non-AMI patients. The blue square represents gene expression in the AMI group, and the red circle represents gene expression in the healthy control group.
